# Predictive factors of body weight loss in patients with type 2 diabetes treated with GLP-1 receptor agonists: a 52-week prospective real-life study

**DOI:** 10.3389/fendo.2025.1674308

**Published:** 2025-09-25

**Authors:** Alfredo Vozza, Domenico Triggiani, Margherita Fanelli, Giuseppe Lisco, Deborah Coletto, Carlo Custodero, Sara Volpe, Davide Racaniello, Valentina Colaianni, Valentina Lavarra, Rosselia Maggipinto, Andrea Portacci, Cosimo Tortorella, Antonio Moschetta, Giuseppina Piazzolla

**Affiliations:** ^1^ Interdisciplinary Department of Medicine, University of Bari “Aldo Moro”, Bari, Italy; ^2^ Department of Precision and Regenerative Medicine and Ionian Area, University of Bari “Aldo Moro”, Bari, Italy; ^3^ Department of Translational Biomedicine and Neuroscience, University of Bari “Aldo Moro”, Bari, Italy

**Keywords:** GLP-1 receptor agonists, predictive factors, body weight loss, metformin, type 2 diabetes, fat mass, skeletal muscle mass, hepatic steatosis

## Abstract

**Introduction:**

Glucagon-like peptide-1 receptor agonists (GLP-1RAs) are widely prescribed for their efficacy in glycemic control and weight reduction, but patient response is heterogeneous and predictors of weight loss remain insufficiently defined. This 52-week prospective, observational study aimed to identify predictors of weight reduction (≥5% from baseline) in patients with type 2 diabetes mellitus (T2D) undergoing GLP-1RA therapy (semaglutide or dulaglutide, including oral formulations).

**Methods:**

A total of 194 adults with T2D initiating GLP-1RA therapy were evaluated at baseline, and after 6, and 12 months of therapy. To identify predictors of weight loss, variables differing between Responders (weight loss ≥5% than baseline) and Non-Responders were evaluated by ROC analysis and tested in univariate and multivariate logistic regression models adjusted for age, gender, GLP-1RA type and dosage.

**Results:**

At 6 and 12 months, 58% and 49% of patients, respectively, achieved the primary outcome. Responders at 12 months exhibited elevated BMI, waist circumference, hepatic steatosis indices, fat mass, and insulin levels at baseline, along with reduced muscle-to-fat and muscle-to-visceral adipose tissue ratios. Moreover, female gender, younger age, shorter disease duration, and non-use of metformin prior to enrollment were significantly associated with response. Notably, early response at 6 months strongly predicted 12-month success.

**Conclusions:**

Our results highlight a valuable interplay between body composition, liver involvement, and the incretin response, also suggesting a maximal synergistic effect between metformin and GLP-1RAs when treatments are initiated concurrently rather than sequentially. These data provide valuable insights for the development of individualized treatment strategies.

## 1 Introduction

Over the last decades, obesity and type 2 diabetes (T2D) have emerged as two of the most pressing global health challenges not only due to their growing prevalence but also because of a tight pathophysiological interconnection between the two conditions. The term “diabesity” is employed to reflect a complex metabolic disorder characterized by insulin resistance, glucose impairment, low-grade chronic inflammation, obesity, altered body composition, and increased cardiovascular risk. Importantly, diabesity is also strongly associated with premature cardiovascular diseases, including coronary artery disease and, most notably, heart failure in younger individuals, which is rapidly becoming a major public health concern worldwide due to the sharp rise in obesity, diabetes, and related metabolic conditions (1).

Within this context, body weight loss has become a key therapeutic target, as even modest reductions (5 to 10%) can lead to substantial improvements in glycemic control, lipid profiles, liver steatosis, and systemic inflammation (2). However, individual responses to weight-loss therapies vary widely among individuals with T2D and obesity.

Glucagon-like peptide-1 receptor agonists (GLP-1RAs) are medications used to achieve integrated management of diabetes and obesity, and show good results in terms of glucose control, cardio-renal protection and weight loss (3–5). Agents such as liraglutide, dulaglutide, subcutaneous and oral semaglutide, have demonstrated significant effects not only on HbA1c reduction but also on body weight loss (5), through mechanisms involving delayed gastric emptying, central modulation of appetite and satiety, and improved insulin and leptin signaling (6, 7). Beyond their established metabolic effects, GLP-1RAs have also recently emerged as promising therapeutic options for metabolic dysfunction-associated steatotic liver disease (MASLD), a condition for which no specific pharmacological treatments were available until very recently (8, 9).

Randomized controlled trials (RCTs) have consistently demonstrated the efficacy of GLP-1RAs in promoting clinically meaningful weight loss, with average reductions ranging from 5% to 15% depending on the agent and study population (10–13). Nevertheless, in real-world settings, some patients fail to achieve significant weight loss with GLP-1RAs, despite adequate titration of the agent and appropriate compliance to prescription, and the ability to identify responders beforehand is still limited. Thus, identifying predictors of weight loss has become a key research objective.

To date, only a limited numbers of investigations have attempted to identify predictors of weight loss response, mostly focused on isolated anthropometric (e.g., Body Mass Index-BMI), demographic (e.g., gender), metabolic (e.g., glycated hemoglobin-HbA1c, lipids), hormonal (e.g., insulin, leptin, ghrelin) parameters, or concurrent medications, but the emerging data still appear conflicting (5, 14, 15). Moreover, despite the strong pathophysiological rationale linking hepatic steatosis and body composition abnormalities to obesity and T2D, no previous studies have explored their prognostic role in predicting weight loss response to GLP-1RAs.

In this context, we conducted a 52-week prospective observational study aimed at identifying clinical, biochemical, ultrasound (US) and bioimpedance parameters as possible predictors of weight loss in a cohort of patients with T2D.

## 2 Materials and methods

### 2.1 Study design, institution, and ethics

This was a 52-week, prospective, observational, real-life study conducted at Metabolic Disorders Outpatients Clinic of the Department of Internal Medicine, University of Bari “Aldo Moro” (Italy) in accordance with the general ethical principles for medical research on humans inspired by the Declaration of Helsinki (16). The study protocol was formally approved by the Ethics Committee of the University of Bari (n. 6468 version 2_amendment of August 4, 2022).

### 2.2 Screening for eligibility of study participants

The study included patients aged ≥18 years with established T2D, who were prescribed a GLP-1RA (once-weekly subcutaneous semaglutide or dulaglutide, up to the maximum available dose of 1 mg or 1.5 mg, respectively, or daily oral semaglutide up to the maximum dose of 14 mg) as part of their standard clinical management. Treatment selection and prescribed dosages were driven by current guidelines and clinical recommendations.

Exclusion criteria included type 1 diabetes, pregnancy or lactation, ongoing or previous use of GLP-1RAs, ongoing treatment with Sodium-Glucose Cotransporter 2 Inhibitors (SGLT2i) or other antihyperglycemic medications affecting body composition other than metformin, history of bariatric surgery, very-low or low-calorie ketogenic dietary regimens during the previous 12 months, recent hospitalization (<3 months), Hepatitis B or C viral infection, ethanol consumption (>30 grams/day in men and >20 grams/day in women), any malignancies, and conditions interfering with body composition assessment (e.g., implantable electronic devices such as cardioverter defibrillators or pacemakers, or limb amputation).

### 2.3 Study protocol

Patients were consecutively screened between September 2022 and February 2024. During this period, a total of 850 patients with T2D were evaluated for eligibility. Eligible patients were fully informed about the study purposes and provided written informed consent to participate. Finally, a total of 194 patients with T2D was included in the study. Enrolled patients underwent baseline (T0), 6-month (T6), and 12-month (T12) evaluations comprising anthropometric measurements, biochemical tests, bioimpedance analysis, and US assessment.

Clinical and anthropometric assessments included smoking and alcohol habits, office blood pressure, heart rate, body weight (BW), waist circumference (WC), and BMI. Laboratory evaluations comprised complete blood count, lipid profile, fasting glucose, HbA1c, serum creatinine with estimated glomerular filtration rate (eGFR), Aspartate Aminotransferase (AST), Alanine Aminotransferase (ALT), Gamma-Glutamyl Transferase (γGT), uric acid, fasting insulin, and C-peptide. Body composition was measured by phase-sensitive, octopolar bioimpedance analysis (Seca mBCA 525, Seca GmbH & Co., KG, Hamburg, Germany) as previously described (17), providing phase angle, Total Body Water (TBW), Extracellular Water (ECW), Skeletal Muscle Mass (SMM), Skeletal Muscle Index (SMI), fat mass index (FMI), Fat-Free Mass Index (FFMI), and Visceral Adipose Tissue (VAT). Liver steatosis was assessed by ultrasonography (Logiq E9; GE Healthcare) using a semiquantitative score (0–3) (18). Derived indices included HOMA-IR for insulin resistance, the Hepatic Steatosis Index (HSI) (19) and the Fatty Liver Index (FLI) (20) for liver steatosis, the Fibrosis-4 index (Fib-4) (21) and the AST-to-platelet ratio index (APRI) (22) for liver fibrosis. Finally, handgrip strength (HG) was evaluated with a hydraulic dynamometer (Lafayette Instrument, Lafayette, IN, USA), and the Muscle Quality Index (MQI) was calculated as the ratio of SMM to HG. All patients received standardized lifestyle recommendations, including advice on adopting a low-carbohydrate diet and increasing their weekly physical activity by 100 minutes, spread across 4–5 sessions per week. All participants agreed to follow these recommendations. However, adherence to lifestyle interventions was not systematically monitored during the study period, reflecting the real-life nature of this observational research.

Due to missing data, the initial cohort of 194 individuals decreased to 188 at T6 and 158 at T12. Given the very low proportion of patients lost to follow-up at T6 (3.1%), no specific analyses were performed for these cases. In contrast, the higher attrition rate at T12 (18.6%) prompted a dedicated comparison of baseline characteristics between completers and non-completers to assess potential attrition bias. The reasons for loss to follow-up were systematically recorded and categorized as therapy discontinuation (gastrointestinal side effects: n=2, patient preference n=6), incomplete assessments for technical issues (inability to perform abdominal ultrasound or bioimpedance analysis: n=16), lack of adherence to scheduled visits (n=12).

### 2.4 Study outcomes and endpoints

The primary study outcome was a change in body weight from baseline to 6 and 12 months of GLP-1RA treatment. More precisely, patients were categorized as “Responders” (R) if they reached the threshold of ≥5% weight loss from baseline at follow-up; otherwise they were considered “Non Responders” (NR). Changes in glucometabolic parameters (fasting glycemia, HbA1c, HOMA-index, lipid levels), body composition (paying particular attention to FM, SMM and VAT changes), biochemical (AST, ALT, GGT, FLI, HSI, APRI score, Fib-4) and US signs of Metabolic dysfunction-Associated Steatotic Liver Disease (MASLD) over time were also evaluated.

The primary endpoint of the study was the identification of independent predictors of achieving ≥5% body weight loss at follow-up, among a broad set of anthropometric, biochemical, US, and bioimpedance variables assessed at baseline.

**Figure 1:**
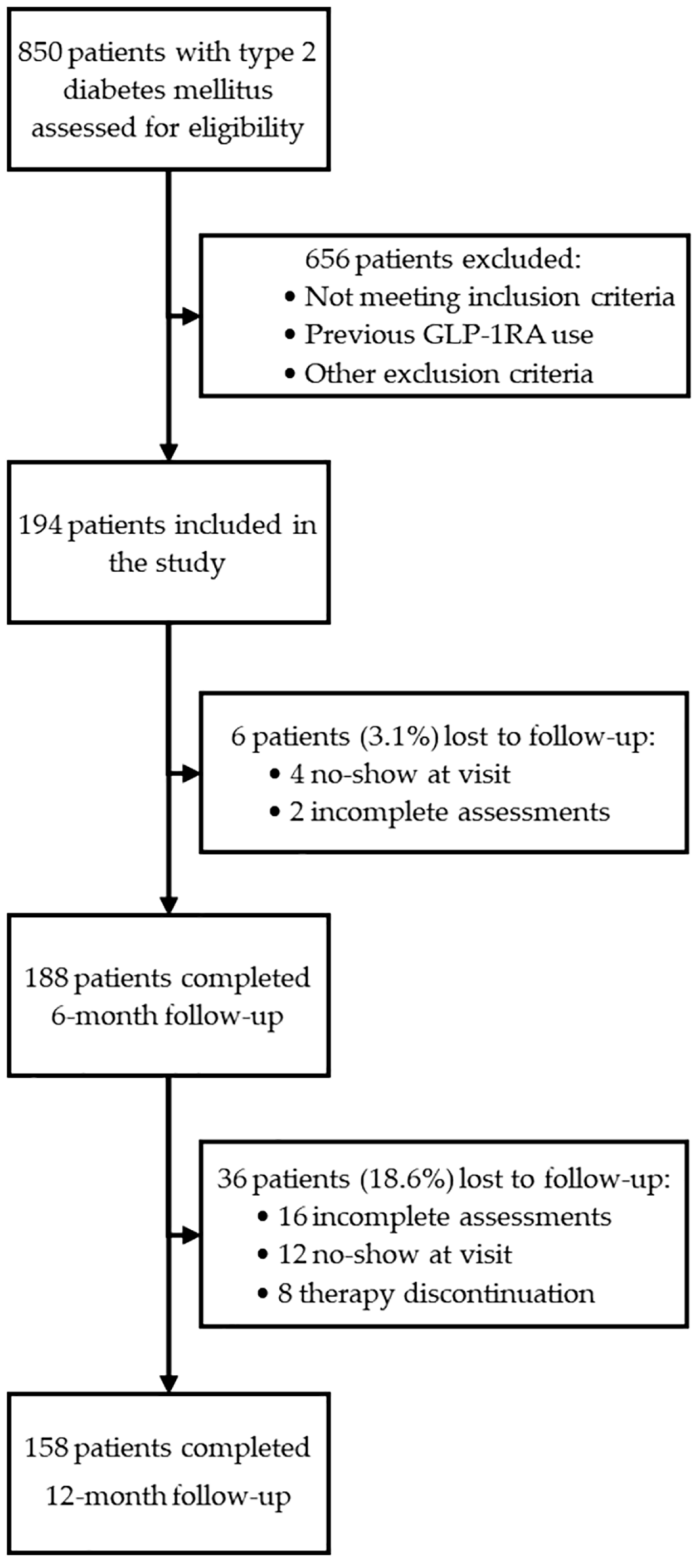
Flow diagram illustrating the screening, inclusion, and follow-up of patients enrolled in the study. A total of 850 individuals with type 2 diabetes mellitus were assessed for eligibility. After applying inclusion and exclusion criteria, 194 patients were enrolled and received GLP-1RA therapy. Complete follow-up data were available for 188 patients (96.9%) at 6 months and for 158 patients (81.4%) at 12 months.

A flow chart illustrating the selection process and follow-up of the study population is presented in **Figure 1**.

### 2.5 Statistical analysis

Continuous variables were described using mean, standard deviation, median, minimum, and maximum values. Their distribution was assessed using the Shapiro-Wilk test to inform the appropriate use of parametric or non-parametric statistical tests. Categorical variables were reported as frequencies and percentages.

Differences in continuous variables over time (T0, T6, T12) were analyzed using linear mixed-effects models with fixed effects for time and random intercepts for subjects, which allowed the inclusion of both normally and non-normally distributed variables and accounted for within-subject variability. For categorical variables, McNemar’s test was applied to assess changes over time.

To identify independent predictors of achieving ≥5% body weight reduction at 6 and 12 months (primary outcome), we first compared baseline characteristics between R and NR using univariate analysis (T-tests or Mann–Whitney U for continuous variables, chi-square or Fisher’s exact test for categorical variables).

All baseline variables that showed significant differences between R and NR were further evaluated using Receiver Operating Characteristic (ROC) curve analysis. This approach had two main objectives: to select accurate predictors (AUC > 60% and p < 0.05) and to determine the optimal cut-off values using the Youden index. These thresholds were used to dichotomize continuous variables for subsequent analysis.

All variables derived through this process were then tested independently in logistic regression models adjusted for age, gender, type and dosage of GLP-1RA, using the respective weight loss outcome at 6 and 12 months as the dependent variable. Given that metformin naive status was not randomized, and could have been influenced by baseline clinical characteristics, additional multivariable logistic regression models were constructed to rigorously assess its independent predictive value. These models were adjusted not only for age, gender, GLP-1RA type and dosage, but also for other key predictors including baseline BMI, waist circumference, fat mass index, GGT, fatty liver index, insulin, or duration of diabetes, in order to account for potential confounding and isolate the effect of concurrent metformin initiation.

To assess the potential impact of attrition bias, baseline demographic, clinical, and biochemical characteristics were compared between patients completing and not completing the 12-month follow-up. Variables differing significantly between groups were not included in further analyses.

All analyses were performed using IBM SPSS Statistics, version 27.0.1.0 (IBM Corp., Armonk, NY, USA). A two-sided p-value <0.05 was considered statistically significant.

A power analysis was performed using GPower version 3.1 to assess the adequacy of the sample size for the primary endpoint. The analysis was based on a total sample of 180 participants, a two-sided alpha of 0.05 and an R² of 0.10 between predictors and covariates. Across a range of the clinical odds ratios, the statistical power consistently exceeded 80% for each of them, confirming the robustness of the sample size in detecting meaningful associations with the primary outcome.

## 3 Results

### 3.1 Baseline characteristics of the study population

The baseline characteristics of the study population are summarized in **Table 1**.

**Table 1:** Baseline characteristics of the study population.

Variable	Cohort (N = 194)
Age (years), mean ± SD	63 ± 9.9
Male, n (%)	98 (50.5)
Disease duration (years), median (IQR)	4 (1 - 10)
History of smoking, n (%)	111 (57.2)
Pack-years, median (IQR)	4.5 (0 - 20)
Body Weight (kg), median (IQR)	85 (73 - 91.1)
BMI (kg/m²), median (IQR)	31.2 (28.1 - 33.8)
Waist circumference (cm), median (IQR)	107 (99 - 113)
Systolic BP (mmHg), median (IQR)	130 (120 - 140)
Diastolic BP (mmHg), median (IQR)	80 (70 - 85)
Heart rate (bpm), median (IQR)	72.5 (68 - 80)
Handgrip (kg), mean ± SD	30.6 ± 9.9
Hemoglobin (g/dL), mean ± SD	13.7 ± 1.5
Hematocrit (%), mean ± SD	41.3 ± 4.02
Platelets (x10^3^/uL), median (IQR)	242 (195.5 - 287.5)
Creatinine (mg/dL), median (IQR)	0.8 (0.7 - 1)
eGFR (ml/min), median (IQR)	88.5 (73.5 - 99.5)
Urea (mg/dL), median (IQR)	37 (30.5 - 45)
Uric acid (mg/dL), median (IQR)	5.0 (4.2 - 5.9)
Glucose (mg/dL), median (IQR)	116 (104 - 137.5)
HbA1c (mmol/mol), median (IQR)	47 (41.5 - 54)
C-peptide (ng/mL), median (IQR)	2.9 (2.2 - 3.5)
Insulin (μU/mL), median (IQR)	12.4 (7.8 - 17.9)
HOMA-IR, median (IQR)	3.6 (2.2 - 5.2)
TG/HDL, median (IQR)	2.3 (1.6 - 4.1)
GGT (U/L), median (IQR)	32 (22 - 50.5)
AST (U/L), median (IQR)	20 (18 - 25)
ALT (U/L), median (IQR)	29 (20.8 - 40.7)
HSI, median (IQR)	45.6 (42.3 - 49.8)
FLI, median (IQR)	83.4 (60.7 - 90.5)
APRI score, median (IQR)	0.2 (0.2 - 0.3)
Fib-4, median (IQR)	1.1 (0.8 - 1.4)
Total cholesterol (mg/dL), median (IQR)	154 (133.5 - 176.5)
Triglycerides (mg/dL), median (IQR)	114 (89.5 - 159.5)
LDL cholesterol (mg/dL), median (IQR)	80 (60 - 101.5)
HDL cholesterol (mg/dL), median (IQR)	47 (40 - 57)
Metformine-Naïve, n (%) ^a^	26 (13.6)
Ultrasonographic evidence of liver steatosis, n (%)	176 (90.7)

Data are expressed as absolute values and percentage for categorical variables and as mean ± SD or median ± interquartile range for continuous variables. a: data available for 191 patients.

BMI, body mass index; BP, blood pressure; eGFR, estimated glomerular filtration rate; HbA1c, glycated hemoglobin; HOMA-IR, Homeostasis Model Assessment of Insulin Resistance; TG, triglycerides; HDL, high-density lipoprotein; GGT, Gamma-Glutamyl Transferase; AST, Aspartate Aminotransferase; ALT, Alanine Aminotransferase; HSI, Hepatic Steatosis Index; FLI, Fatty Liver Index; APRI, AST to Platelet Ratio Index; Fib-4, Fibrosis-4; LDL, low-density lipoprotein.

A total of 194 patients were enrolled at the end of the recruitment period. The patients’ gender ratio was comparable (98 males, 96 females), approximately 1:1. The median duration of disease was relatively brief (4 years) and 30 of the enrolled patients were newly diagnosed (duration 0 years). Sixty patients (30.9%) were prescribed once-weekly (qw) subcutaneous semaglutide; after the starting dose of 0.25 mg qw for 4 weeks, they continued with 0.5 mg, with 20% of patients increasing treatment to 1 mg qw by the sixth month. Thirty-six patients (18.6%) were prescribed dulaglutide (41.7% at 0.75 mg and 58.3% at 1.5 mg qw). Ninety-eight patients (50.5%) were prescribed oral semaglutide; after the starting daily dose of 3 mg for 30 days, almost all patients increased to 7 mg per day from the second month (99%) and only 1% further increased to 14 mg/day by the sixth month. It should be noted that insulin therapy at enrollment was reported in only three patients (two in the oral semaglutide group and one in the dulaglutide group), all of whom were on 10 IU of insulin glargine and maintained the same regimen throughout the follow-up.

The population showed a marked metabolic burden, characterized by high values of the HOMA-IR and triglyceride/HDL-cholesterol ratio. The cohort was predominantly composed of overweight or frankly obese patients with a high median waist circumference. However, it is worth noting that 14 normal-weight patients were also enrolled in the study.

The non-invasive indices of hepatic steatosis, HSI and FLI, were suggestive of substantial MASLD, which was confirmed by ultrasound. In particular, 90.7% of the patients had US signs of hepatic steatosis, and most of them (66.9%) showed moderate or severe steatosis.

### 3.2 Multiparameter effects of GLP-1RA therapy

Significant improvements in fasting glycemia, HbA1c, body weight, BMI, waist circumference, and systolic blood pressure, were already evident after the first 6 months and confirmed at T12 compared to baseline (**Table 2**).

**Table 2:** Changes in clinical, anthropometric and biochemical parameters after 6 (T6) and 12 (T12) months of GLP-1RA therapy compared to baseline (T0).

Parameter	δ T6 vs T0	δ T12 vs T0
Body Weight (kg)	-6 ± 0.4**	-6.29 ± 0.49**
BMI (kg/m²)	-2.2 ± 0.2**	-2.37 ± 0.18**
Waist Circumference (cm)	-4.6 ± 0.5**	-5.99 ± 0.53**
Systolic BP (mmHg)	-7.5 ± 1.4**	-3.36 ± 1.38*
Diastolic BP (mmHg)	-3.3 ± 0.5**	-1.04 ± 0.92
Heart rate (bpm)	2 ± 0.7**	-0.54 ± 0.74
Handgrip (kg)	-1 ± 0	-1.18 ± 0.58*
Creatinine (mg/dL)	0.02 ± 0.01	0.001 ± 0.92
eGFR (mL/min/1.73m²)	-2.19 ± 0.81**	-0.93 ± 0.89
Glucose (mg/dL)	-19.49 ± 1.91**	-19.35 ± 2.04**
HbA1c (mmol/mol)	-7.65 ± 0.79**	-7.52 ± 0.83**
C-peptide (ng/mL)	-0.11 ± 0.13	0.03 ± 0.13
Insulin (µU/mL)	-2.60 ± 0.20**	-1.54 ± 0.91
HOMA-IR	-1.55 ± 0.35**	-1.23 ± 0.34**
TG/HDL	-0.31 ± 0.11**	-0.45 ± 0.11**
GGT (U/L)	-2.67 ± 4.09	-2.03 ± 4.52
AST (U/L)	-2.45 ± 0.86**	-1.67 ± 0.88
ALT (U/L)	-5.61 ± 1.63**	-3.37 ± 1.75*
HSI	-2.83 ± 0.41**	-2.4 ± 0.48**
FLI	-10.96 ± 1.11**	-12.75 ± 1.21**
APRI	-0.03 ± 0.01**	-0.01 ± 0.01
Fib-4	0.02 ± 0.03	0.05 ± 0.04
Total cholesterol (mg/dL)	-22.01 ± 2.8**	-24.61 ± 2.77**
Triglycerides (mg/dL)	-14.59 ± 3.52**	-18.19 ± 3.83**
LDL cholesterol (mg/dL)	-17.27 ± 2.36**	-22.44 ± 2.48**
HDL cholesterol (mg/dL)	-1.56 ± 0.72*	1.25 ± 0.79

Data are expressed as mean ± standard error (SE) of the change from baseline. *p<0.05 vs T0; **p<0.01 vs T0.

BMI, Body Mass Index; BP, Blood Pressure; eGFR, Estimated Glomerular Filtration Rate; HbA1c, Glycated Hemoglobin; HOMA-IR, Homeostasis Model Assessment of Insulin Resistance; TG, triglycerides; HDL, high-density lipoprotein; GGT, Gamma-Glutamyl Transferase; AST, Aspartate Aminotransferase; ALT, Alanine Aminotransferase; HSI, Hepatic Steatosis Index; FLI, Fatty Liver Index; APRI, AST to Platelet Ratio Index; Fib-4, Fibrosis-4; LDL, Low-Density Lipoprotein.

In contrast, the significant reduction in diastolic blood pressure and the increase in heart rate observed at T6 compared to T0 were no longer relevant after 12 months of treatment (**Table 2**). Renal function tests (serum creatinine and eGFR) remained substantially stable from T0 to T12. Between T6 and T12, GLP-1RA dosages were further adjusted according to clinical response and tolerability. In particular, 60.3% of patients treated with subcutaneous semaglutide maintained the 0.5 mg dose qw, while 39.7% received 1 mg qw after the sixth month. Among patients on dulaglutide, 36.1% remained on 0.75 mg qw, and 63.9% received 1.5 mg. Finally, in the group treated with oral semaglutide, 82.1% were on 7 mg/day, and 17.9% received 14 mg per day, by the end of the follow-up.

Insulin resistance indices (HOMA-IR, triglyceride/HDL ratio), serum levels of total and HDL cholesterol, triglycerides, ALT, and steatosis indices (HSI, FLI) improved significantly throughout the follow-up, while AST and APRI scores were found to be reduced compared to baseline only at T6 (**Table 2**).

**Figure 2:**
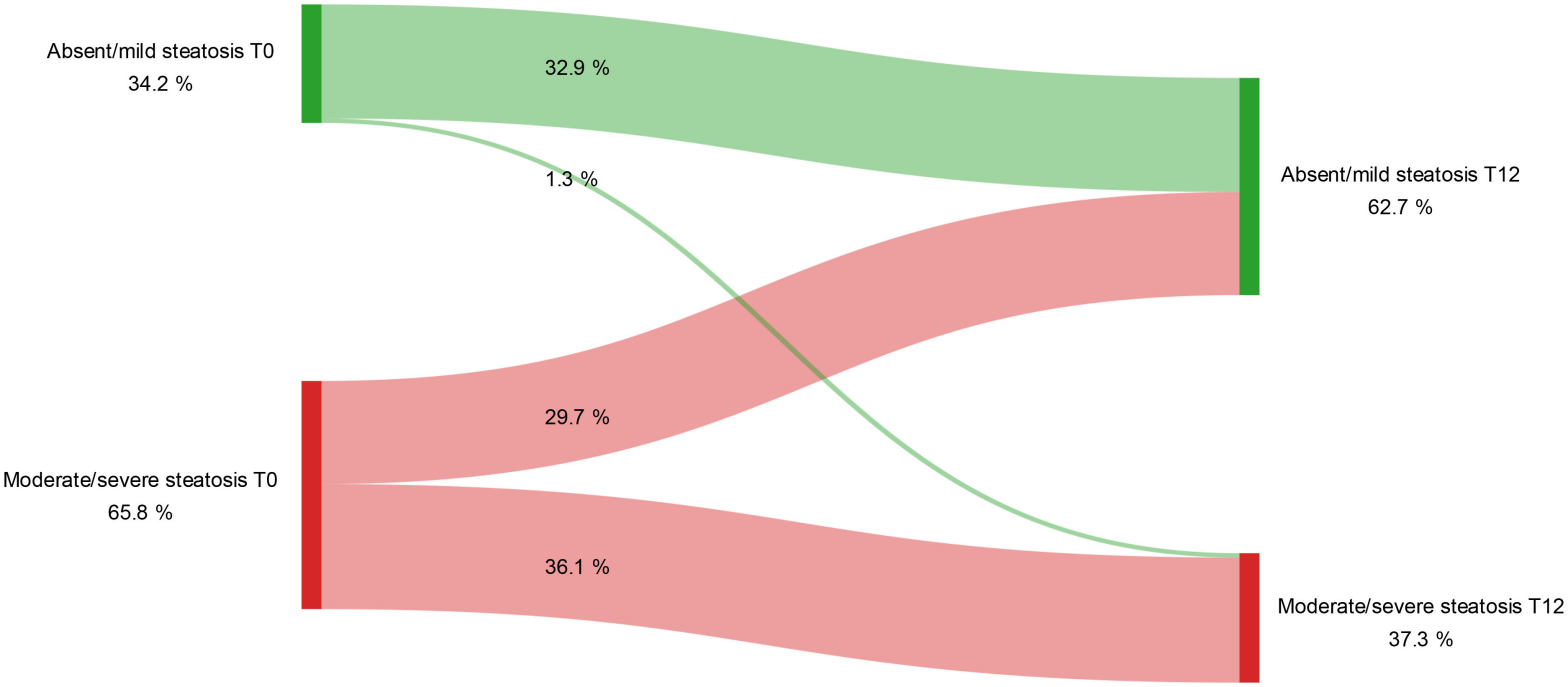
Variation in the degree of hepatic steatosis detected by ultrasound during the study period. Longitudinal changes in degrees of hepatic steatosis from baseline (T0) to 12 months (T12) of GLP-1RA treatment (McNemar’s test T0 vs T12: p < 0.001). Steatosis was categorized as either absent/mild or moderate/severe based on ultrasonographic assessment at each time point.

The beneficial effects of GLP-1RAs on MASLD were also supported by US data. Specifically, the number of patients with moderate to severe steatosis decreased gradually, with significant reductions observed between baseline and the end of the follow-up period (**Figure 2**). These changes were confirmed by McNemar’s test (T0 vs T6: p = 0.001; T6 vs T12: p = 0.021).

Finally, as previously observed (23–25), GLP-1RAs exerted a significant impact on body composition, mainly by inducing a predominant reduction in Fat Mass (FM) and Visceral Adipose Tissue (VAT) compared to fat-free mass compartment. In fact, although Skeletal Muscle Mass (SMM) was also reduced after 12 months of treatment, in addition to FM, there was still a significant increase over baseline in both the SMM/VAT and SMM/FM ratios, indicating that patients lost more visceral fat and fat mass than muscle mass (**Table 3**).

**Table 3:** Changes in bioimpedance parameters after 6 (T6) and 12 (T12) months of GLP-1RA therapy compared to baseline (T0).

Parameter	T0	δ T6 vs T0	δ T12 vs T0
FM (kg)	34.69 ± 12.00	-5.66 ± 0.44**	-4.7 ± 0.47**
FM (%)	39.57 ± 8.35	-4.28 ± 0.37**	-2.97 ± 0.39**
FMI (kg/m²)	13.05 ± 4.81	-2.11 ± 0.16**	-1.78 ± 0.18**
FFM (kg)	52.04 ± 11.28	0.12 ± 0.27	-1.15 ± 0.28**
FFM (%)	60.43 ± 8.35	4.28 ± 0.37**	2.97 ± 0.39**
FFMI (kg/m²)	19.22 ± 3.15	0.004 ± 0.1	-0.42 ± 0.10**
SMM (kg)	24.15 ± 6.39	-0.33 ± 0.16*	-0.99 ± 0.17**
SMI (kg/m²)	8.85 ± 1.87	-0.12 ± 0.06*	-0.35 ± 0.06**
MQI (kg/kg)	1.29 ± 0.34	-0.02 ± 0.03	-0.02 ± 0.03
SMM/FM (kg/kg)	0.73 ± 0.27	0.17 ± 0.03**	0.09 ± 0.02**
VAT (L)	4.22 ± 2.48	-0.63 ± 0.08**	-0.72 ± 0.09**
SMM/VAT (kg/L)	6.94 ± 3.70	1.38 ± 0.3**	1.26 ± 0.31**
Resistance (Ω)	504.60 ± 82.95	-11.5 ± 4.19**	10.84 ± 4.25*
Reactance (Ω)	47.82 ± 9.21	-1.16 ± 0.48*	0.21 ± 0.53
Phase Angle (°)	5.47 ± 0.79	-0.05 ± 0.04	-0.15 ± 0.04**
TBW (L)	38.87 ± 8.35	0.06 ± 0.23	-0.84 ± 0.24**
ECW (L)	17.75 ± 3.70	-0.04 ± 0.12	-0.48 ± 0.13**
ECW/TBW (%)	45.74 ± 4.50	0.22 ± 0.31	0.26 ± 0.32

Data are expressed as mean ± standard error (SE). *p<0.05 vs T0; **p<0.01 vs T0.

FM, Fat Mass; FMI, Fat Mass Index; FFM, Fat Free Mass; FFMI, Fat Free Mass Index; SMM, Skeletal Muscle Mass; SMI, Skeletal Muscle Index; MQI, Muscle Quality Index; VAT, Visceral Adipose Tissue; TBW, Total Body Water; ECW, Extracellular Body Water.

### 3.3 Predictors of body weight loss response

The response to GLP-1RA therapy, in terms of body weight (BW) loss, was assessed separately at 6 and 12 months, using a BW loss ≥5% from baseline as the clinical threshold to define R. The cohort was therefore stratified into R and NR at each timepoint, and their baseline characteristics were compared.

Of note, due to missing data at T12, first of all we compared baseline characteristics between completers and non-completers patients at T12, and we found that only systolic blood pressure, creatinine, eGFR, HSI, and Fib-4 score showed significant differences between the two groups. Therefore, these variables were excluded from subsequent analyses to avoid potential confounding (**Supplementary Table S1**). All other variables were comparable, including gender (p=0.94), history of smoking (p=0.88), US presence of liver steatosis (p=0.89), liver steatosis grade (p=0.99), concomitant metformin use (p=0.42), the type (p=0.20) and dosage of GLP-1RA prescribed (p=0.07), thereby reducing the likelihood of systematic attrition bias.

#### 3.3.1 Quantitative predictors of body weight loss response

Baseline differences between quantitative variables of patients classified as R or NR after 6 and 12 months of GLP-1RA therapy are shown in **Table 4**.

**Table 4:** Baseline differences in clinical, anthropometric, biochemical, and body composition quantitative variables between Responders (R) and Non-Responders (NR) after 6 (T6) and 12 months (T12) of GLP-1RA therapy.

Parameter	T6 (n=188)			T12 (n=158)		
	R (58%)	NR (42%)	P	R (49%)	NR (51%)	P
Duration of diabetes (years)	6.7 ± 0.8	6.8 ± 0.8	ns	5.7 ± 0.8	8.5 ± 1	0.002
Age (years)	63 ± 0.9	62.8 ± 1.15	ns	61.6 ± 1	65.7 ± 1.1	0.008
BMI (kg/m²)	33.4 ± 0.6	31.6 ± 0.8	0.01	34.3 ± 0.8	29.3 ± 0.6	<0.001
Waist circumference (cm)	111.3 ± 1.3	107.1 ± 1.8	0.01	112.4 ± 1.6	102.8 ± 1.5	<0.001
Insulin (µU/mL)	16.8 ± 1.3	14.4 ± 1.4	ns	19.3 ± 1.7	11.7 ± 0.9	<0.001
HOMA-IR	5.4 ± 0.6	4.3 ± 0.4	ns	6.2 ± 0.7	3.4 ± 0.3	<0.001
C-peptide (ng/mL)	3.2 ± 0.1	3.3 ± 0.3	ns	3.5 ± 0.2	2.7 ± 0.1	0.002
GGT (U/L)	58.7 ± 10.2	33.1 ± 2.2	0.01	51.6 ± 7.3	43.7 ± 12.6	0.004
ALT (U/L)	39.9 ± 3	30.3 ± 1.7	ns	38.6 ± 3	31.2 ± 2.8	0.016
FLI	80.2 ± 2.1	70.5 ± 3	0.009	80.9 ± 2.1	64.5 ± 3.4	<0.001
Fat Mass (kg)	36.9 ± 1.2	32.8 ± 1.5	0.006	38.5 ± 1.4	27.8 ± 1.2	<0.001
Fat Mass (%)	41.3 ± 0.8	37.7 ± 1	0.003	42.2 ± 0.8	35 ± 1.1	<0.001
FMI (kg/m²)	13.9 ± 0.5	12.3 ± 0.6	0.006	14.6 ± 0.6	10.2 ± 0.5	<0.001
FFM (%)	58.6 ± 0.8	62.3 ± 1	0.003	57.7 ± 0.8	65 ± 1.1	<0.001
SMM/FM (kg/kg)	0.7 ± 0.02	0.8 ± 0.05	0.007	0.67 ± 0.02	0.96 ± 0.07	<0.001
SMM/VAT (kg/L)	6.2 ± 0.2	7.7 ± 0.6	0.04	6.2 ± 0.3	8.6 ± 0.7	0.003
VAT (L)	4.3 ± 0.2	4 ± 0.3	ns	4.5 ± 0.3	3.5 ± 0.2	0.04

Data are expressed as mean ± standard error (SE); “ns” indicates non-significant difference (p > 0.05). Statistical comparisons refer to baseline values.

BMI, Body Mass Index; HOMA-IR, Homeostasis Model Assessment of Insulin Resistance; GGT, Gamma-Glutamyl Transferase; ALT, Alanine Aminotransferase; HSI, Hepatic Steatosis Index; FLI, Fatty Liver Index; FMI, Fat Mass Index; FFM, Fat Free Mass; SMM, Skeletal Muscle Mass; FM, Fat Mass; VAT, Visceral Adipose Tissue.

At T6, 58% of the cohort had achieved the predefined weight loss outcome of ≥5%, while at T12, the percentage of responders was 49%.

As can be seen, most parameters became relevant as distinguishing features at baseline between R and NR only when the response was assessed at T12, although some parameters were able to characterize R subjects already after the first 6 months of treatment (BMI, WC, GGT, FLI, FM, FMI, FFM%, SMM/FM, and SMM/VAT).

**Figure 3:**
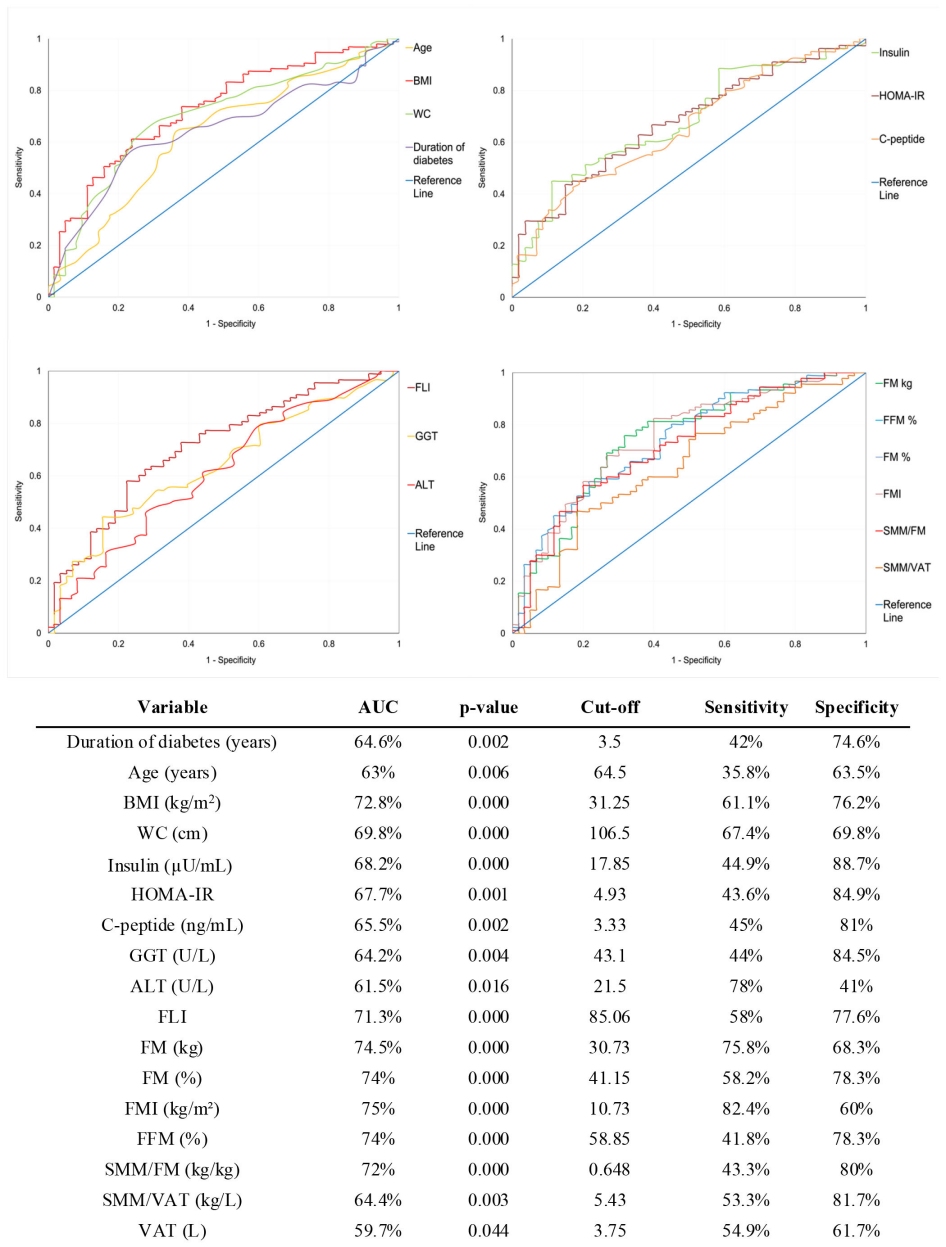
Receiver Operating Characteristic (ROC) curves for baseline predictors of weight loss response at 12 months. The optimal cut-off points for each variable were identified using Youden’s Index. Sensitivity and specificity values correspond to the optimal cut-off points. AUC, Area Under the Curve; BMI, Body Mass Index; WC, Waist Circumference; HOMA-IR, Homeostasis Model Assessment of Insulin Resistance; GGT, Gamma-Glutamyl Transferase; ALT, Alanine Aminotransferase; FLI, Fatty Liver Index; FM, Fat Mass; FMI, Fat Mass Index; FFM, Fat-Free Mass; SMM, Skeletal Muscle Mass; VAT, Visceral Adipose Tissue.

To further investigate the predictive value of these continuous variables, we performed ROC curve analyses to assess their discriminative capacity at T6 (data not shown) and T12 (**Figure 3**).

Variables with poor performance (AUC < 60% or p > 0.05) were excluded from further evaluation. For the remaining variables, the optimal cut-off point was determined using Youden’s index and used to dichotomize each variable.

To identify independent predictors of body weight loss response to GLP-1RA therapy, each dichotomized variable was entered into a separate logistic regression model adjusted for age, gender, type and dosage of GLP-1RA used. Several baseline variables were found as independent predictors of the primary outcome at T6 months: BMI ≥ 28.5 kg/m2 (OR 2.1; 95% CI: 1.01–4.3; p = 0.046), GGT ≥ 45.5 U/L (OR 2.3; 95% CI 1.1-5.2; p = 0.034 and waist circumference ≥ 103.5 cm (OR 2.1; 95% CI: 1.03–4.1; p = 0.041).

**Figure 4:**
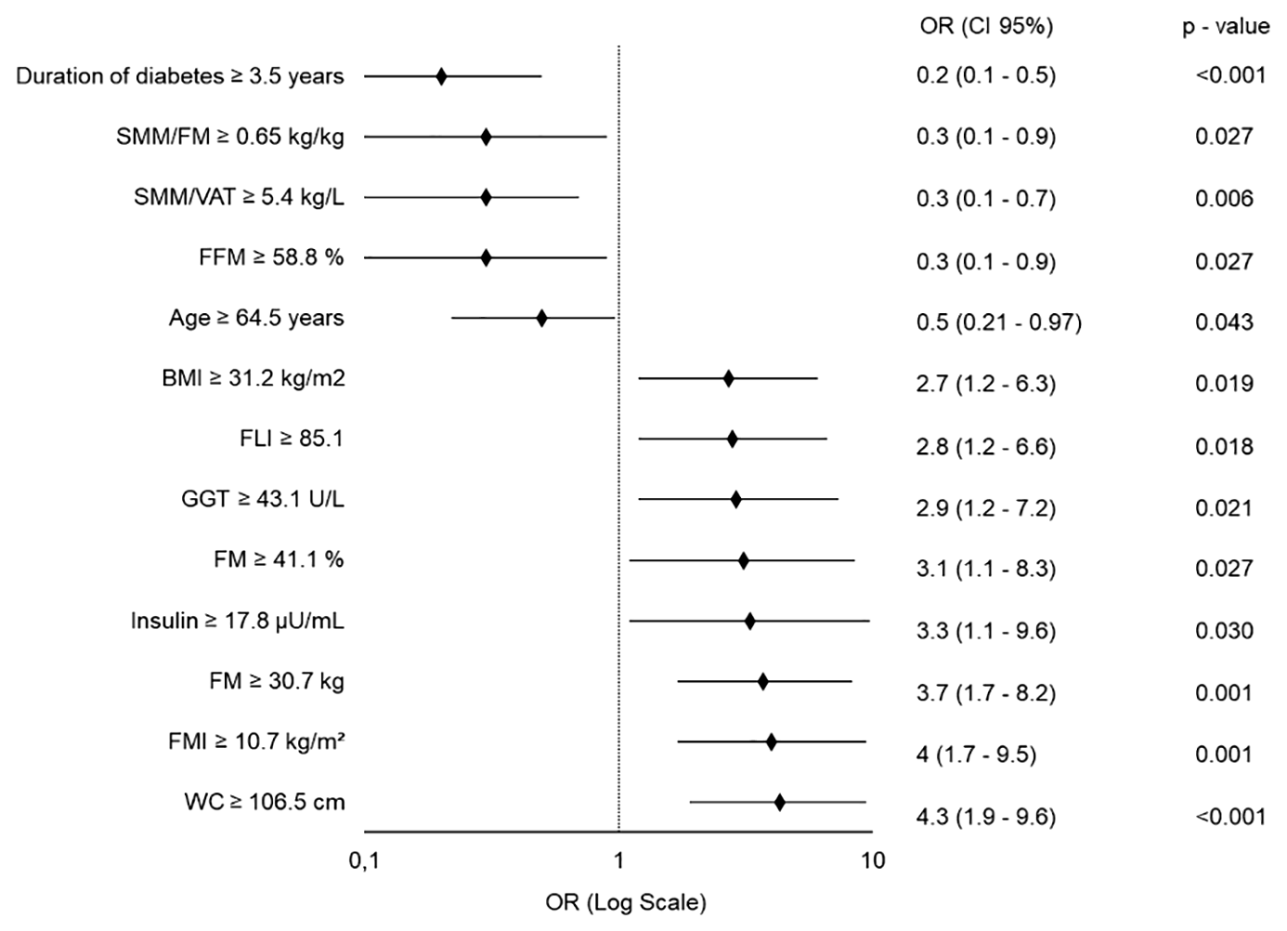
Forest Plot of Quantitative Predictors for ≥5% Body Weight Loss at 12 Months. Each variable was independently tested in a separate binary logistic regression model, adjusted for age, gender (except when analyzing age, adjusted for gender only), type and dosage of GLP-1RA used. All predictors are modeled as dichotomous variables. OR, Odds Ratio; CI, Confidence Interval; SMM, Skeletal Muscle Mass; FM, Fat Mass; VAT, Visceral Adipose Tissue; FFM, Fat-Free Mass; BMI, Body Mass Index; FLI, Fatty Liver Index; GGT, Gamma-Glutamyl Transferase; FMI, Fat Mass Index; WC, Waist Circumference.

When considering patients classified as R at T12, the predictive model identified a broader set of baseline variables significantly associated with long-term body weight loss response (**Figure 4**). Specifically, duration of diabetes, age, FFM%, and the SMM/FM and SMM/VAT ratios were inversely associated with response, whereas higher values of the other variables increased the likelihood of achieving ≥5% body weight loss (see **Figure 4**).

#### 3.3.2 Qualitative predictors of body weight loss response

No significant differences emerged between patients classified as R or NR after the first 6 months of therapy, regarding baseline gender distribution, smoking status (current or past), presence or the degree of steatosis detected by ultrasound. The only qualitative variable that showed a significant association with the outcome was non-use of metformin at baseline, as 84.6% of metformin-naïve patients were classified as R at T6, compared to 53.9% of those already taking metformin at the start of GLP-1RA therapy (χ² = 8.6; p = 0.003).

When the outcome was evaluated after 12 months, analysis of baseline qualitative variables revealed additional parameters significantly associated with response to GLP-1RAs. Metformin-naïve status at baseline was confirmed to be associated with the outcome, as 91% of metformin-naïve patients achieved a weight loss ≥5% than baseline, compared to 54% of those already on metformin before the study enrolment (χ² = 11.2, p = 0.04). A significant gender difference was also observed, since 72% of women were classified as R versus 49% of men (χ² = 8.7, p = 0.003). Importantly, a significant trend was identified between the baseline degree of hepatic steatosis and the primary outcome. The proportion of R increased progressively along with the steatosis severity, as R were 36.4% in patients without steatosis, 55.5% in grade 1, 60% in grade 2, and 78.8% in grade 3, respectively. While the overall chi-square test approached significance (χ² = 7.68, p = 0.053), the linear-by-linear association confirmed a statistically significant trend (χ² for linear trend = 7.00, p= 0.008), suggesting that more severe hepatic involvement might predict a greater likelihood of achieving weight loss ≥5%.

Given the significant associations observed between non-use of metformin at baseline, female gender, steatosis, and the achievement of the outcome after 12 months of GLP-1RA therapy, we further investigated whether baseline metformin use was related to any of these variables. No significant associations were found between metformin use at baseline and gender (χ² = 0.76, p = 0.38), the presence of hepatic steatosis (χ² = 0.54, p = 0.46), or the degree of steatosis when evaluated as an ordinal variable (χ² for linear trend = 2.00, p = 0.16). Moreover, there was no association between ongoing metformin intake at T6 and the achievement of responder status at T6 (χ² = 0.432, p-value = 0.51), nor between metformin intake at T12 and responder status at T12 (χ² = 0.60, p-value = 0.44). Conversely, starting metformin concurrently with GLP-1RA therapy at T0 was significantly associated with responder status both at T6 (χ² = 4.76, p-value = 0.029) and T12 (χ² = 6.07, p-value = 0.014), highlighting a potential synergistic effect when both therapies are initiated simultaneously.

Finally, early response to therapy at T6 was found to be associated with long-term response. In fact, among patients who had already achieved ≥5% weight loss after 6 months of treatment, 84.3% maintained the response at T12 while only 30.2% of NR at T6 showed a significant weight loss at T12 (χ² = 45.77, p < 0.001).

To assess the prognostic value of baseline qualitative variables with treatment response, we constructed separate binary logistic regression models at both 6 and 12 months, adjusting each model for age, gender (or only age, when the variable was gender), dosage and type of GLP-1RA. Variables with a significant linear trend, such as steatosis grade, were included despite non-significant global associations, based on clinical relevance.

**Figure 5:**
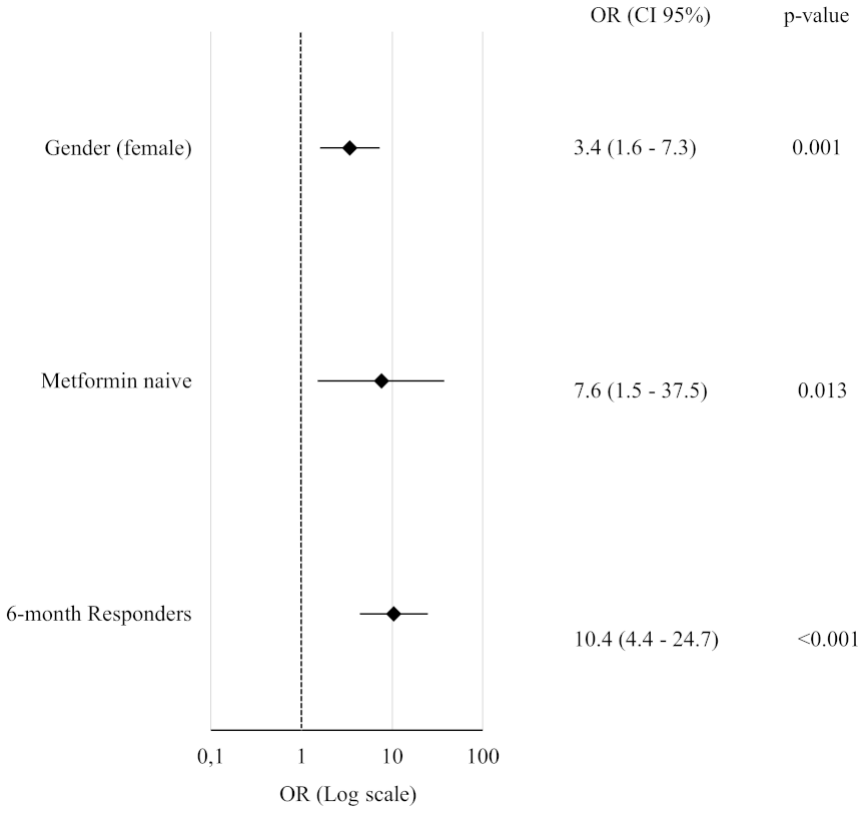
Forest Plot of Qualitative Predictors for ≥5% Body Weight Loss at 12 Months. Each variable was tested in a separate binary logistic regression model adjusted for age, gender (except when analyzing gender itself, which was only adjusted for age), type and dosage of GLP-1RA. All odds ratios (OR) are reported with corresponding 95% confidence intervals and p-values.

At 6 months, baseline metformin intake was confirmed to be a negative predictor of response, with metformin-naïve patients showing a higher likelihood of achieving ≥5% weight loss (OR = 4; 95% CI: 1.3–12.8; p = 0.018). Consistently, as illustrated in **Figure 5**, the independent predictive value for the 12-month outcome was confirmed for most of the qualitative variables that significantly differed between R and NR at baseline.

Notably, metformin-naïve status retained its predictive role even in fully adjusted models including BMI (p = 0.021), FMI (p = 0.036), insulin (p = 0.028), FLI (p = 0.033), GGT (p = 0.021), diabetes duration (p = 0.014) or waist circumference (p = 0.022).

Similarly, significant weight loss after 6-months of therapy maintained its strong predictive value for long term weight loss or maintenance after further adjustment for BMI and diabetes duration (OR = 10.5; 95% CI: 4.4–25.2; p<0.001).

### 3.4 Exploratory analysis: predictors of ≥10% weight loss at 12 months

An additional analysis was performed by setting the outcome threshold at a more stringent criterion, namely a 10% body weight loss after 12 months. In this context, 29.7% of patients achieved this outcome. Logistic regression analysis adjusted for age, gender, dosage and type of GLP-1RA revealed that positive predictors of ≥10% body weight loss were the female gender (OR 3.2; 95% CI: 1.5–6.8; p = 0.003), FLI ≥ 85.1 (OR 3.4; 95% CI: 1.4–8.3; p = 0.008), WC ≥ 106.5 cm (OR 4.6; 95% CI: 1.9–11.1; p < 0.001), BMI ≥ 31.2 kg/m2 (OR 4.0; 95% CI: 1.6–10.0; p = 0.003), and metformin-naïve status (OR 7.1; 95% CI: 2.4–21.4; p < 0.001).

## 4 Discussion

In recent years, RCT and real-world data have demonstrated the multiple benefits of GLP-1RAs in T2D. In particular, their effects on body weight are consistent regardless of the molecule utilized and route of administration (5), but interindividual variability is a clinical concern as not all individuals achieve satisfactory weight loss, stressing the need for predictive tools helping clinicians to guide therapeutic decisions.

In this study, we investigated predictors of weight loss response to GLP-1RAs over a 12-month follow-up. Unlike many RCTs, we adopted a pragmatic approach, focusing on real-life patients and selecting a ≥5% body weight reduction as the primary outcome, in line with established clinical relevance. Due to the real-life nature of the study, the analyses have also been adjusted for the type and dosage of GLP-1RA used to take into account their difference in efficacy and minimize potential confounding.

We found that R to GLP-1RAs had an elevated BMI and waist circumference at baseline, consolidating the role of anthropometric characteristics as predictors of clinical response and their significance in therapeutic decision-making (5, 26). In our cohort, individuals with a baseline BMI ≥31.2 kg/m² had a 2.7-fold higher likelihood of achieving the predefined weight loss response at T12, compared to those with lower BMI values. Similarly, a waist circumference ≥106.5 cm was associated with a 4.3-fold increased probability of response.

Demographic factors, specifically gender and age, emerged as significant predictors of weight loss at 12 months. Female gender was associated with a significantly greater response, with women exhibiting a 3.5-fold higher probability of achieving the outcome at T12, compared to men. The result is consistent with recent evidence indicating a better response of women to GLP-1RAs (5, 27). This effect may be partly attributed to hormonal differences and variations in fat distribution that modulate drug responsiveness. In line with the meta-analysis by Wong et al. (5), age emerged as a negative predictor of weight loss, reflecting the decline in metabolic adaptation due to aging. Furthermore, we found that a longer duration of diabetes (≥3.5 years) was associated with five-fold lower probability of achieving clinically meaningful weight loss. This finding suggests that early-stage diabetes, potentially characterized by a greater metabolic flexibility and residual beta-cell function, may favor a more robust response to GLP-1RA therapy.

The substantial heterogeneity in the individual response to GLP-1RAs in term of weight loss is still a pending issue and has been the focus of a recent review by Saturnino et al. (28), who analyzed RCTs, real-world evidence, cohort studies, and systematic reviews conducted between 2016 and 2024, aimed at identifying predictors of weight loss among GLP-1RA users. Only high HbA1c levels at baseline emerged as a negative predictor of weight loss (29), while no clear predictive role was found for BMI or gender and, in some studies, responders had even a lower body weight at baseline than non-responders (30). A possible negative effect of insulin resistance and insulin treatment on the efficacy of GLP-1RAs was hypothesized but not confirmed. The apparent discrepancies with our data may be explained by substantial differences in the study populations. In fact, the studies reviewed by Saturnino et al. were focused on individuals with obesity (BMI >30) regardless of the presence of T2D, including adolescents, whereas our study investigated an adults-only diabetic population with no BMI restrictions.

A pivotal aspect of our study was the composite evaluation of body composition and hepatic steatosis parameters on the primary outcome. While body fat distribution and visceral adiposity have long been hypothesized as modulators of therapeutic response, our study is the first to evaluate their predictive role. Specifically, fat mass and fat mass index emerged as significant predictors of weight loss, reinforcing the role of adipose tissue as a central determinant of insulin resistance and systemic inflammation, two intimately connected processes that represent key targets of GLP-1RA therapy. In parallel, GGT and a simple hepatic steatosis index, namely FLI, were strongly associated with a favorable treatment response to GLP-1RAs. These findings are not coincidental but interrelated as also liver steatosis is a specific hallmark of metabolic dysfunction in T2D. Consistently, elevated fasting insulin level was also found to be a strong predictor of weight loss response, establishing hyperinsulinemia and insulin resistance as key biochemical markers of the underlying shared dysfunction.

In this context, the importance of careful assessment of basal body composition is also underscored by the negative predictive value of skeletal muscle to fat mass or visceral adipose tissue ratio. As previously reported (23–25), our data show that GLP-1RAs clearly induce “healthy” weight loss mainly related to a more pronounced reduction in FM and VAT rather than SMM. In light of the metabolic protective role of SMM in T2D (31), the results of our study support the safety of GLP-1RAs and should lead to a refinement of the therapeutic target in subjects with a high SMM to adipose tissue ratio, beyond misleading high BMI values.

An additional relevant predictor that emerged from our analyses was the use of metformin at baseline. Unexpectedly, patients naïve to metformin were seven times more likely to achieve the outcome after 12 months of GLP-1RAs than those already taking metformin before starting GLP-1RA. The independent predictive value of metformin-naive status at baseline was rigorously confirmed after adjustment for the main confounders (gender, age, type and dosage of GLP-1RA) and other key predictors, including BMI at baseline, waist circumference, FMI, GGT, FLI, insulin or diabetes duration. It should also be noted that, in our cohort, almost all metformin-naïve patients started metformin concomitantly with GLP-1RA, and this simultaneous initiation was strongly associated with achieving the weight loss outcome. This suggests a synergistic effect of metformin and GLP-1RA on weight loss response through a simultaneous rather than sequential approach. Both metformin and GLP-1RAs are known to improve leptin sensitivity, making the brain more responsive to the anorexigenic effects of the adipokine (32–36). However, Bensignor et al. recently demonstrated that individuals with a lower leptin response showed greater weight loss under GLP-1RA treatment (37). This supports the hypothesis that metformin exposure preceding the initiation of GLP-1RAs could paradoxically reduce their efficacy on weight loss by improving basal leptin sensitivity. This pharmacodynamic interplay supports the concept of a transient window of enhanced incretin responsiveness, which could be strategically exploited in therapeutic planning.

Finally, our study highlights that the achievement of weight loss ≥5% at 6 months represents the strongest predictor of sustained weight loss at 12 months, increasing by tenfold the likelihood of reaching the final outcome, in line with what has recently been described in adolescents with obesity (38). This has relevant clinical implications in routine practice and reinforces the importance of close follow-up in the first months of GLP-1RA therapy as a guide to individualized treatment planning. Notably, the robustness of our findings was further confirmed when the outcome threshold was raised to a more stringent ≥10% body weight loss at 12 months, as the main predictors retained their statistical significance.

This study has several limitations that should be acknowledged. Firstly, despite the adequate sample size, which allowed for robust statistical analyses and clinically meaningful insights, it was conducted at a single center. This design may limit the generalizability of the findings to other populations and settings, underlining the need for multicenter validation. Secondly, although all participants were given standardized recommendations on adopting a low-carbohydrate diet and increasing physical activity at enrollment, lifestyle adherence was only self-reported and not systematically monitored. The lack of objective assessments of diet and exercise may have introduced potential confounding, and future studies should incorporate validated questionnaires or digital monitoring tools to better capture these factors. Additional exploratory subgroup analyses would also have been of interest but were limited by the consequent reduction in sample size. In particular, we attempted stratified analyses by GLP-1RA type (dulaglutide, weekly semaglutide, oral semaglutide), but the number of patients in each subgroup was insufficient to provide adequately powered results. To minimize this potential source of bias, all multivariable models were adjusted for both type and dosage of GLP-1RA. Similarly, with regard to concomitant antidiabetic therapies, only metformin was adequately represented and could therefore be evaluated as a potential modifier, whereas the limited use of insulin (only 3 patients) did not allow meaningful analysis. Moreover, our study did not explore the influence of genetic or epigenetic factors, which could also contribute to inter-individual variability in weight loss response to GLP-1RAs. However, recent large-scale investigations have begun to address this challenge, with results suggesting no significant associations between GLP-1RA-induced weight loss and currently known genetic factors (39). Notably, although our study was designed with a 12-month follow-up, which provided robust information on medium-term outcomes, it did not allow assessment of the long-term sustainability of weight loss with GLP-1RAs, an aspect that remains crucial for clinical practice. Finally, approximately 18% of patients were considered non-completers at T12, but baseline characteristics were largely comparable between completers and non-completers. To minimize potential confounding and reduce the likelihood that attrition bias significantly influenced the study results, the few variables that differed were not included in the models used to investigate predictive factors.

## 5 Conclusions

Our study provides an evidence-based framework for anticipating therapeutic outcomes in patients with T2D treated with GLP-1RA, beyond the restrictive inclusion criteria of RCTs. These results highlight the interplay among body composition, liver involvement, and the incretin response, providing valuable insights for the development of individualized treatment strategies.

## Data Availability

The raw data supporting the conclusions of this article will be made available by the authors, without undue reservation.

## Supplementary material

Supplementary Table 1 Baseline characteristics of participants completing and not completing the 12-month follow-up. Data are presented as mean ± standard deviation (SD) for normally distributed variables or median (interquartile range, IQR) for non-normally distributed variables. Continuous variables were compared using the independent-samples *t*-test or the Mann–Whitney *U* test, depending on the distribution. BMI, body mass index; BP, blood pressure; WBC, white blood cells; eGFR, estimated glomerular filtration rate; U-ACR, urinary albumin-to-creatinine ratio; HbA1c, glycated hemoglobin; HOMA-IR, Homeostasis Model Assessment of Insulin Resistance; TG, triglycerides; HDL, high-density lipoprotein; GGT, Gamma-Glutamyl Transferase; AST, Aspartate Aminotransferase; ALT, Alanine Aminotransferase; HSI, Hepatic Steatosis Index; FLI, Fatty Liver Index; APRI, AST to Platelet Ratio Index; Fib-4, Fibrosis-4; LDL, low-density lipoprotein; SMM, Skeletal Muscle Mass; VAT, Visceral Adipose Tissue; FM, Fat Mass; MQI, Muscle Quality Index; SMI, Skeletal Muscle Index; FMI, Fat Mass Index; FFM, Fat Free Mass; FFMI, Fat Free Mass Index; TBW, Total Body Water; ECW, Extracellular Body Water.
