# Supplementary material for: Predictive factors of body weight loss in patients with type 2 diabetes treated with GLP-1 receptor agonists: a 52-week prospective real-life study
Source: Front Endocrinol (Lausanne). 2025 Sep 25;16:1674308. doi: 10.3389/fendo.2025.1674308 (PMC12507565; doi:10.3389/fendo.2025.1674308)
Supplement: Supplementary Table 1 — Baseline characteristics of participants completing and not completing the 12-month follow-up. Data are presented as mean ± standard deviation (SD) for normally distributed variables or median (interquartile range, IQR) for non-normally distributed variables. Continuous variables were compared using the independent-samples t-test or the Mann–Whitney U test, depending on the distribution. BMI, body mass index; BP, blood pressure; WBC, white blood cells; eGFR, estimated glomerular filtration rate; U-ACR, urinary albumin-to-creatinine ratio; HbA1c, glycated hemoglobin; HOMA-IR, Homeostasis Model Assessment of Insulin Resistance; TG, triglycerides; HDL, high-density lipoprotein; GGT, Gamma-Glutamyl Transferase; AST, Aspartate Aminotransferase; ALT, Alanine Aminotransferase; HSI, Hepatic Steatosis Index; FLI, Fatty Liver Index; APRI, AST to Platelet Ratio Index; Fib-4, Fibrosis-4; LDL, low-density lipoprotein; SMM, Skeletal Muscle Mass; VAT, Visceral Adipose Tissue; FM, Fat Mass; MQI, Muscle Quality Index; SMI, Skeletal Muscle Index; FMI, Fat Mass Index; FFM, Fat Free Mass; FFMI, Fat Free Mass Index; TBW, Total Body Water; ECW, Extracellular Body Water. [file Table1.docx]

**Table S1. Baseline characteristics of participants completing and not completing the 12-month follow-up**

| **Variable** | **Non completers at T12**  **(n =36)** | **Completers at T12**  **(n = 158)** | **p-value** |
| --- | --- | --- | --- |
| Age (years), mean ± SD | 62 ± 12.1 | 63.2 ± 9.4 | 0.572 |
| Disease duration (years), median (IQR) | 9 (4 – 13.5) | 3 (1 - 10) | 0.184 |
| Pack-years, median (IQR) | 20 (7.5 – 27.7) | 1.8 (0 - 18.1) | 0.413 |
| Body Weight (kg), median (IQR) | 86 (75.2 – 94.6) | 84.7 (72.6 - 93.9) | 0.160 |
| BMI (kg/m²), median (IQR) | 32.8 (29.1 – 38.7) | 30.8 (28.3 - 34.5) | 0.109 |
| Waist circumference (cm), median (IQR) | 110 (106 – 123.5) | 106 (97.7 - 113.2) | 0.099 |
| Systolic BP (mmHg), median (IQR) | 135 (120 **–** 145) | 130 (120 - 140) | **0.026** |
| Diastolic BP (mmHg), median (IQR) | 80 (67.5 – 90) | 80 (70 - 86.2) | 0.610 |
| Heart rate (bpm), median (IQR) | 75 (66 – 80.5) | 72.5 (65 - 84) | 0.632 |
| Handgrip (kg), mean ± SD | 28.3 ± 9.4 | 31.1 ± 10 | 0.255 |
| Hemoglobin (g/dL), mean ± SD | 13.6 ± 1.6 | 13.8 ± 1.5 | 0.619 |
| Hematocrit (%), mean ± SD | 41 ± 4.5 | 41.4 ± 3.9 | 0.568 |
| Platelets (/mm³), median (IQR) | 240 (183.5 – 304.5) | 223 (185.2 - 258.5) | 0.072 |
| Creatinine (mg/dL), median (IQR) | 0.77 (0.63 – 0.90) | 0.85 (0.70 – 1.01) | **0.011** |
| eGFR (ml/min), median (IQR) | 96.3 (84 – 104.5) | 83.5 (71.5 - 100) | **0.010** |
| Urea (mg/dL), median (IQR) | 38 (34 – 46) | 37(28.7 - 45.7) | 0.141 |
| Uric acid (mg/dL), median (IQR) | 4.7 (3.9 – 6.5) | 4.9 (4.3 - 6) | 0.513 |
| Glucose (mg/dL), median (IQR) | 106 (89 – 157.5) | 116 (105.7 - 140.5) | 0.438 |
| HbA1c (mmol/mol), median (IQR) | 46 (39.5 – 58.5) | 47.5 (41 - 54) | 0.749 |
| C-peptide (ng/mL), median (IQR) | 2.5 (2 – 3.6) | 2.9 (2.4 - 3.6) | 0.841 |
| Insulin (μU/mL), median (IQR) | 14 (9.3 – 18.6) | 11.7 (8.7 - 18.2) | 0.846 |
| HOMA-IR, median (IQR) | 3.6 (2.4 – 6.2) | 3.4 (2.4 - 5.2) | 0.570 |
| TG/HDL, median (IQR) | 2.4 (1.5 – 2.9) | 2.1 (1.4 - 4.3) | 0.805 |
| GGT (U/L), median (IQR) | 23 (17 – 40.5) | 32.5 (22 - 51.7) | 0.538 |
| AST (U/L), median (IQR) | 20 (17.2 – 27) | 22.1 (19 - 26.2) | 0.363 |
| ALT (U/L), median (IQR) | 24 (19 – 46.5) | 32 (24.7 - 46.5) | 0.078 |
| HSI, median (IQR) | 46 (41.1 – 54.9) | 45.2 (42.6 - 50.5) | **0.006** |
| FLI, median (IQR) | 83.1 (60.1 – 94.8) | 80.2 (55.2 - 90.1) | 0.076 |
| APRI score, median (IQR) | 0.2 (0.19 – 0.35) | 0.27 (0.21 - 0.35) | 0.059 |
| Fib-4, median (IQR) | 1.1 (0.79 – 1.6) | 1.13 (0.83 - 1.4) | **0.041** |
| Total cholesterol (mg/dL), median (IQR) | 129 (106 – 183.5) | 150.5 (129.7 - 168) | 0.533 |
| Triglycerides (mg/dL), median (IQR) | 97 (92 – 124.5) | 105.5 (78.5 - 167.5) | 0.932 |
| LDL cholesterol (mg/dL), median (IQR) | 71 (41.5 – 93.5) | 75.5 (55.2 - 101.2) | 0.573 |
| HDL cholesterol (mg/dL), median (IQR) | 42 (38 – 54.5) | 46 (40 - 56.2) | 0.620 |
| SMM/VAT (kg/L), median (IQR) | 4.6 (3.8 – 6.6) | 6.5 (5.2 - 8.5) | 0.296 |
| SMM/FM (kg/kg), median (IQR) | 0.6 (0.47 – 0.94) | 0.8(0.54 - 0.95) | 0.582 |
| MQI (kg/kg), median (IQR) | 1.3 (1.13 – 1.37) | 1.3 (1.2 - 1.5) | 0.186 |
| SMI (kg/m²), median (IQR) | 8.5 (7.8 – 9.8) | 8.8 (7.8 - 10) | 0.769 |
| FM (kg), median (IQR) | 34.4 (27.8 – 48.1) | 30.8 (26.8 - 35.1) | 0.185 |
| FMI (kg/m²), median (IQR) | 13.4 (10.2 – 19.2) | 11.4 (9.7 - 14.1) | 0.432 |
| FM (%), median (IQR) | 43.7 (33.3 – 48.7) | 37.2 (33.1 - 44.5) | 0.679 |
| FFM (kg), median (IQR) | 52.8 (42.6 – 57.5) | 51 (44 - 59) | 0.671 |
| FFMI (kg/m²), median (IQR) | 18.9 (17.6 – 21.1) | 19 (17 - 20.9) | 0.500 |
| FFM (%), median (IQR) | 56.3 (51.3 – 66.7) | 62.8 (55.4 - 66.8) | 0.679 |
| SMM (kg), median (IQR) | 23.5 (18.7 – 27.5) | 23.7 (20.4 - 28.1) | 0.868 |
| Resistance (Ω), mean ± SD | 500.9 ± 75.2 | 505.4 ± 84.7 | 0.784 |
| Reactance (Ω), mean ± SD | 46.3 ± 9.4 | 48.1 ± 9.2 | 0.316 |
| Phase Angle (°), mean ± SD | 5.3 ± 0.8 | 5.5 ± 0.8 | 0.162 |
| VAT (L), median (IQR) | 4.5 (3.3 – 5.7) | 3.5 (2.4 - 5) | 0.261 |
| TBW (L), median (IQR) | 39.3 (32.7 – 42.5) | 38 (33.5 - 43.4) | 0.562 |
| ECW (L), median (IQR) | 17.4 (16 – 18.6) | 16.9 (15.5 - 19.1) | 0.343 |
| ECW/TBW (%), median (IQR) | 45.9 (44.5 – 48.5) | 44.8 (43.5 - 47.4) | 0.433 |

Data are presented as mean ± standard deviation (SD) for normally distributed variables or median (interquartile range, IQR) for non-normally distributed variables. Continuous variables were compared using the independent-samples *t*-test or the Mann–Whitney *U* test, depending on the distribution.

Abbreviations: BMI=body mass index; BP=blood pressure; WBC=white blood cells; eGFR=estimated glomerular filtration rate; U-ACR=urinary albumin-to-creatinine ratio; HbA1c=glycated hemoglobin; HOMA-IR=Homeostasis Model Assessment of Insulin Resistance; TG=triglycerides; HDL=high-density lipoprotein; GGT=Gamma-Glutamyl Transferase; AST=Aspartate Aminotransferase; ALT=Alanine Aminotransferase; HSI=Hepatic Steatosis Index; FLI=Fatty Liver Index; APRI=AST to Platelet Ratio Index; FIB-4=Fibrosis-4; LDL=low-density lipoprotein; SMM=Skeletal Muscle Mass; VAT=Visceral Adipose Tissue; FM=Fat Mass; MQI=Muscle Quality Index; SMI=Skeletal Muscle Index; FMI=Fat Mass Index; FFM=Fat Free Mass; FFMI=Fat Free Mass Index; TBW=Total Body Water; ECW=Extracellular Body Water..
